# Incarcerated Umbilical Hernia in a 14-Month-Old Female: A Rare Case of Bowel Strangulation

**DOI:** 10.3390/pediatric12030017

**Published:** 2020-10-23

**Authors:** Chrysostomos Kepertis, Maria Tsopozidi, Kleanthis Anastasiadis, Dimitrios Godosis, Charikleia Demiri, Ioannis Spyridakis

**Affiliations:** 2nd Paediatric Surgery Department, “Papageorgiou” General Hospital, Aristotle University of Thessaloniki, Thessaloniki Ring Road, Nea Efkarpia Region, 56403 Thessaloniki, Greece; tmarion88@hotmail.com (M.T.); kaanastasiadis1@gmail.com (K.A.); konpal1453@yahoo.gr (D.G.); harademiri@hotmail.com (C.D.); ispyrida@auth.gr (I.S.)

**Keywords:** umbilical hernia, strangulation, children

## Abstract

An umbilical hernia, although considered a benign condition of childhood, may rarely manifest with serious complications such as incarceration and viscous organ strangulation. One such case is presented in this report in an attempt to enrich the current literature, due to the relative lack of data in regard to complicated umbilical hernias in children and definite guidelines regarding the monitoring and management of uncomplicated cases. In addition, we discuss some of the latest advancements concerning the matter at issue.

## 1. Introduction

An umbilical hernia is a common condition of early childhood, defined as a protrusion of abdominal viscera through a defect in the umbilical ring. An incarcerated hernia is one in which the hernia cannot easily be reduced into the peritoneal cavity, while strangulation occurs when the blood supply to the entrapped tissue is compromised. We present a rare case of umbilical hernia incarceration in a 14-month-old female, which progressed to strangulation of a small bowel loop.

A 14-month-old female presented to the emergency department due to abdominal pain and an episode of vomiting, while clinical examination revealed an irreducible umbilical hernia, with mild discoloration. Parents reported drowsiness and refusal to eat since that morning, accompanied by inability to pass stool for the last three days, which they misinterpreted as occasional constipation.

The child underwent ultrasonographic examination that demonstrated the presence of a hernia at the level of the umbilicus, containing a small bowel loop with adequate motility and perfusion. The width of the abdominal opening was estimated at about 1 cm.

**Figure 1:**
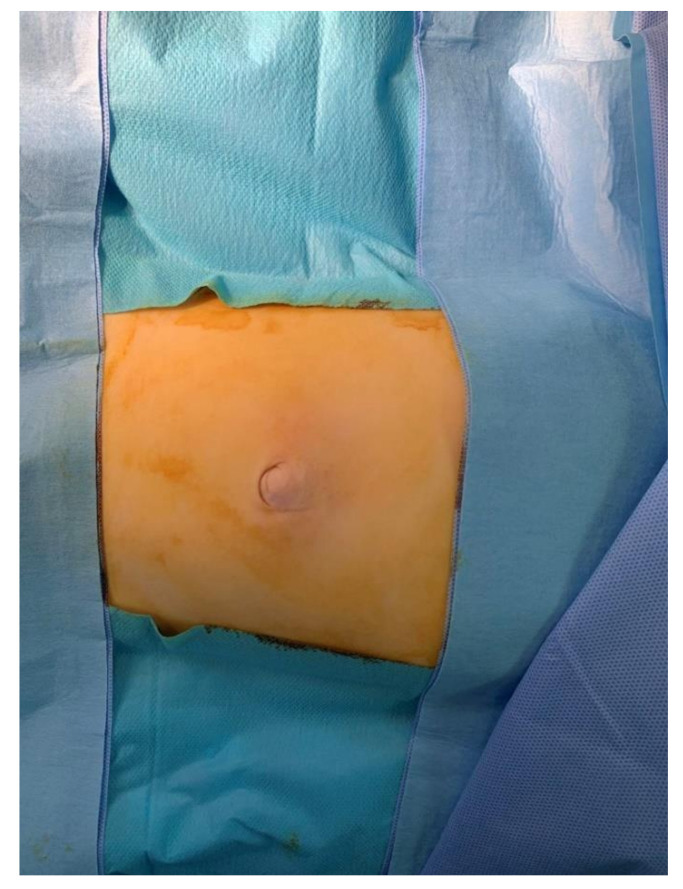
The hernia was not reducible, even after the induction to anesthesia.

**Figure 2:**
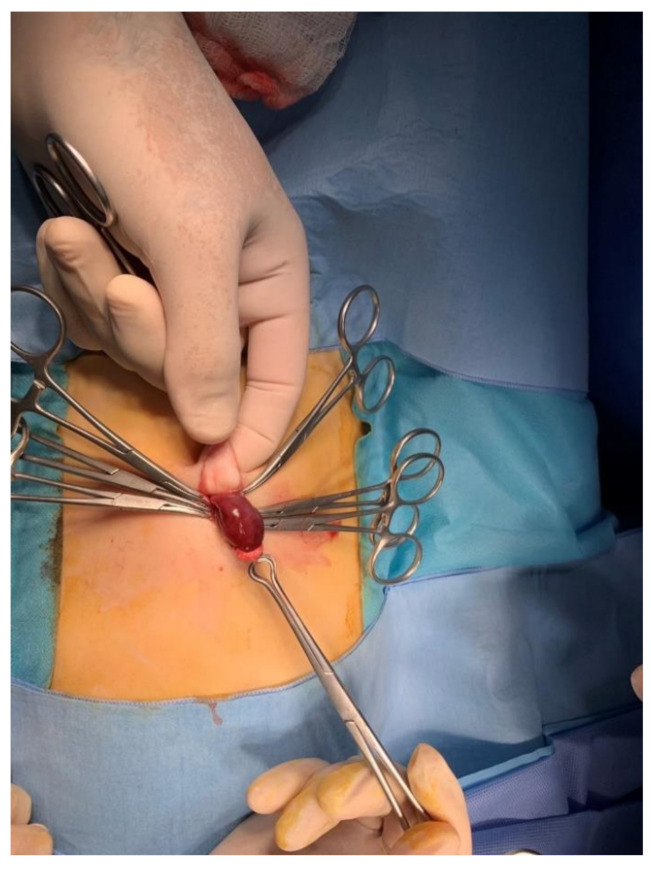
Signs of advanced and irreversible ischemia.

The child was transferred to the operating room shortly after the admission to the pediatric surgery department (Figure 1). The hernia was not reducible even after the induction to anesthesia and the sac was strongly adherent to the umbilicus with the content appearing ischemic, suggesting the chronic nature of the protrusion. After gaining access to the abdominal cavity through a supraumbilical incision, the incarcerated intestine was released. The affected bowel loop—measuring up to 6 cm in length and located 1 m distal to the ligament of Treitz—had signs of advanced and irreversible ischemia (Figure 2). Therefore, an enterotomy with end-to-end anastomosis was performed. The sac was resected and the fascia was closed.

The postoperative course of the child was excellent with early mobilization and satisfactory response to oral feeding, since the child was breastfed in the immediate postoperative period. Following discharge, the patient had an uneventful follow-up.

## 2. Discussion

The incidence of umbilical hernias is about 18–20% in term neonates and around 84% in premature neonates, as it was estimated in an epidemiologic study in Denmark [1]. Some risk factors that probably predispose to this condition are Down’s syndrome, hypothyroidism, prematurity and black ethnicity [2,3,4]. While most cases resolve spontaneously by the age of five, a small percentage of patients present with complications. Incarceration of umbilical hernia in childhood is a rare incident with an overall risk ranging from 0.07% to 0.3% [3], while intestinal strangulation represents an even less common entity. Nonetheless, an incarceration incidence of 40% was observed in Africans [3,4]. According to a recent review by Yoshida et al., the reported cases of incarceration or strangulation worldwide barely amount to 39, including this one [2]. Regardless, in contrast to this study, the patient in our case was a white 14-month-old female, with no history of prematurity, or any other known comorbidities. However, the diameter of the umbilical ring was 1 cm in size, defined as “medium” in the literature, a deciding factor in the development of incarceration [2,5,6]. According to Lassaletta et al., the width of the umbilical ring is considered small when it is less than 0.5 cm, medium between 0.5 and 1.5 cm and large when it is over 1.5 cm [5].

The clinical presentation includes abdominal pain, vomiting, refusal to feeding, inability to pass stool or gas and an irreducible, tender to palpation umbilical bulge, potentially accompanied by discoloration [2,7]. Preverbal children often present with less specific symptoms, such as fussiness, drowsiness, constipation or refusal to eat. Naturally, caregivers mistake those symptoms for other common issues of childhood, like teething, occasional constipation and gastroenteritis, along with others. Therefore, this leads to delayed hospital presentation, as in this case.

So far, the consensus suggests surgical intervention after the age of 4–5 in asymptomatic children, due to spontaneous healing in 85% of patients [1,8,9]. A recent study of 9809 patients, who underwent umbilical hernia repair, concluded that children aged less than 4 years had higher rates of recurrence and unplanned revisits postoperatively [10]. On the other hand, considerable dissatisfaction was observed with the aesthetic outcome in patients undergoing surgery at a higher age [11]. Another study by Sinopidis et al. discussed the involvement of omentum in the pathogenesis of incarceration, a finding that can be used as a diagnostic modality in the detection, via ultrasound, of patients who are more likely to develop complications [12].

## 3. Conclusions

Incarcerated nonreducible umbilical hernias in children require prompt surgical intervention owing to the increased risk of viscous strangulation. Therefore, a high level of alertness should be maintained in regard to children with asymptomatic umbilical hernias. It is imperative for caregivers to have a clear understanding of the condition. Thus, it is crucial that health professionals responsible for the care and follow-up of these children provide parents with all the necessary information, in an effort to avoid morbidity. Further studies are required to determine which children with asymptomatic umbilical hernias are prone to complications and need surgical treatment.
